# Dominant Tree Species and Litter Quality Govern Fungal Community Dynamics during Litter Decomposition

**DOI:** 10.3390/jof10100690

**Published:** 2024-10-03

**Authors:** Wenjing Meng, Lin Chang, Zhaolei Qu, Bing Liu, Kang Liu, Yuemei Zhang, Lin Huang, Hui Sun

**Affiliations:** 1 Collaborative Innovation Center of Sustainable Forestry in Southern China, College of Forestry, Nanjing Forestry University, Nanjing 210037, China; mengwenjing@njfu.edu.cn (W.M.); linchang@njfu.edu.cn (L.C.); qzl941211@njfu.edu.cn (Z.Q.); 202404324@163.com (B.L.); liukang@njfu.edu.cn (K.L.); zymei23394@njfu.edu.cn (Y.Z.); 2 Department of Forest Sciences, Faculty of Agriculture and Forestry, University of Helsinki, 00790 Helsinki, Finland; 3 College of Landscape and Horticulture, Yangzhou Polytechnic College, Yangzhou 225009, China

**Keywords:** litter decomposition, enzyme activity, fungal community function, nutrient cycling, forest types

## Abstract

Litter decomposition is a crucial biochemical process regulated by microbial activities in the forest ecosystem. However, the dynamic response of the fungal community during litter decomposition to vegetation changes is not well understood. Here, we investigated the litter decomposition rate, extracellular enzyme activities, fungal community, and nutrient cycling-related genes in leaf and twig litters over a three-year decomposition period in a pure *Liquidamabar formosana* forest and a mixed *L. formosana*/*Pinus thunbergii* forest. The result showed that during the three-year decomposition, twig litter in the mixed forest decomposed faster than that in the pure forest. In both leaf litter and twig litter, β-cellobiosidase and N-acetyl-glucosamidase exhibited higher activities in the mixed forest, whereas phosphatase, β-glucosidase, and β-xylosidase were higher in the pure forest. The fungal α-diversity were higher in both litters in the pure forest compared to the mixed forest, with leaf litter showing higher α-diversity than twig litter. Fungal species richness and α-diversity within leaf litter increased as decomposition progressed. Within leaf litter, Basidiomycota dominated in the mixed forest, while Ascomycota dominated in the pure forest. Funguild analysis revealed that Symbiotroph and ectomycorrhizal fungi were more abundant in the mixed forest compared to the pure forest. In the third-year decomposition, genes related to phosphorus cycling were most abundant in both forests, with the pure forest having a higher abundance of *cex* and *gcd* genes. Fungal community structure, predicted functional structure, and gene composition differed between the two forest types and between the two litter types. Notably, the fungal functional community structure during the first-year decomposition was distinct from that in the subsequent two years. These findings suggest that dominant tree species, litter quality, and decomposition time all significantly influence litter decomposition by attracting different fungal communities, thereby affecting the entire decomposition process.

## 1. Introduction

In the forest ecosystem, litter decomposition plays important roles in nutrient cycling, linking biogeochemical processes above and below the ground [1,2]. Leaf and other organic matter, including twigs, stems, and propagative structures, accounts for 70% and 30% of aboveground litterfall, respectively [3]. As litter decomposes, soluble compounds leach into the soil, converting organic matter into accessible nutrients for plants [4], thereby maintaining a balance between soil carbon storage and atmospheric CO_2_ release [5]. This process significantly influences forest soil fertility and nutrient cycling, especially microbial nutrient absorption and utilization [6,7].

Litter decomposition is a dynamic process [8] and is influenced by environmental conditions, litter quality, and microbial communities involved in decomposition [4,9]. Different forest types influence the decomposition rate through variations in litter quality. In subtropical forests, the litter decomposition rate rank is as follows: pure *Cinnamomum camphora* forest > mixed *C. camphora* and *Pinus massoniana* forest > pure *P. massoniana* forest [10]. Studies have shown notable variations in decomposition rates among different forest types. For instance, *Populus davidiana*-dominated forests exhibited a higher decomposition rate than *Quercus liaotungensis*-dominated climax forests, primarily due to lower litter quality [11]. Additionally, research indicates that broadleaf forests potentially facilitated litter to decompose faster compared to coniferous forests in British Columbia [12].

Fungi are the main agents in litter decomposition, whereas bacteria participate in the decomposition to a lesser extent in forest ecosystems [13,14]. Fungi are primary decomposers, breaking down organic polymers through the release of hydrolytic and oxidative enzymes [15,16,17]. These fungi frequently encode enzymes such as acidic phosphatase, β-glucosidase, and N-acetylglucosaminidase, while enzymes like hemicellulases or laccase are less common [18]. The extracellular enzyme activities, in turn, could reflect the litter decomposition pattern at a biochemical level in that they are closely associated with element cycling, especially carbon dynamics [19,20]. Significant correlations were observed between mass loss and cellobiohydrolase and polyphenol oxidase activity in a two-year decomposition experiment conducted in Hawaii, USA [21]. Also, a study on leaf litter decomposition in a wet tropical forest reported that 35% of the variance in leaf litter decomposition could be explained by the enzymes [22]. Moreover, from the fungal predicted guild level, saprotrophic fungi can directly modify or decompose recalcitrant lignin [16,23,24], whereas other fungi, including mycorrhizal fungi, pathogens, endophytes, can metabolize organic matter [5,25].

During decomposition, fungal community composition undergoes substratum succession [26]. These changes correspond to evolving catabolic abilities required at different stages of decomposition, influenced by continuous variations in litter quality [27,28,29]. Research indicates that active fungi species richness peaks near the end of the degradation, with ectomycorrhizal fungi becoming the dominant group [30]. Similar microbial succession patterns have been detected in the fungal community during the decomposing of beech leaf litter, where Basidiomycetes dynamically replace Ascomycetes [31]. A 24-month study on decaying *Q. petraea* leaves also confirmed an increase in the abundance of Basidiomycetes over time and enhanced fungal diversity in the fourth month [32]. Research also suggests a strong link between shifts in forest vegetation and changes in fungal communities. The composition of fungal communities in both litter and soil is profoundly modulated by the prevailing tree species, underscoring their significant ecological influence [33,34]. Conifer–broadleaf forests exhibit greater diversity in saprotrophs and other functional groups associated with carbon and nitrogen cycling compared to pure conifer forests [11]. Changes in dominant tree species in mixed boreal forests lead to shifts in soil fungal communities [35], with diverse litter types shaping the saprotrophic fungi group [36,37]. These findings underscore the significance of understanding shifts in fungal communities that correlate with dominant tree species within forest ecosystems.

The original conifer forests are located in Zijin Mountain, Nanjing, China, and have undergone significant successions due to pine wilt disease [38]. Currently, the forest types include mixed broadleaved and coniferous forests dominated by *P. massoniana*, *P. thunbergii*, *Quercus variabilis*, and *Liquidamabar formosana* [39]. Additionally, there are deciduous broadleaved forests dominated by *L. formosana*, *Q. acutissima*, and *Q. fabri* [40]. The transition from pure conifer to mixed conifer and broadleaved forest, and eventually to broadleaved forest, is still ongoing [39,41].

Although changes in fungal communities in different forests are reported frequently, few studies have focused on the internal relationships in the fungal community composition and their actual enzyme productions and gene potentials in the field of litter decomposition. To better understand how fungal communities respond to decomposition time and changes in dominant tree species, we selected a pure broadleaved forest dominated by *L. formosana*, as well as a mixed forest dominated equally by *L. formosana* and *P. thunbergii* to conduct a three-year decomposition experiment. This study tests two hypotheses: (1) decomposition time and changes in dominant tree species influence enzyme activities and fungal community structures during decomposition; and (2) correlations exist between decomposition rate, enzyme activities, and fungal community composition. The findings offer a unique perspective on fungal communities in litter, as most studies focus on microbial communities within the soil.

## 2. Materials and Methods

### 2.1. Study Sites, Litter Bag Embedding, and Sample Collection

The study site (32°16′15′′ N, 118°48′00′′ W) is situated in the Linggu Temple Scenic Area at the southeastern foot of Zijin Mountain in Nanjing, Jiangsu, China. Zijin Mountain rises 448.9 m and covers an area of 2970 hm^2^. This area has a subtropical monsoon climate, with an annual precipitation of 1000–1050 mm and an average temperature of 15.4 °C [42,43].

In this study, we selected two sites: one mixed forest dominated by *L. formosana* and *P. thunbergii* and one pure broadleaved forest dominated by *L. formosana*, situated more than 500 m apart. To avoid self-correlation, we selected three subplots (50 × 80 m) in each forest, located at a distance of 50 m away from each other. In each subplot, we randomly chose three *L. formosana* trees which were good in health status as the sampling trees, therefore generating nine sample trees in the mixed forest and nine sample trees in the pure forest. We assessed the litter degradation by using the traditional mesh bag method [44]; the detailed process is as follows. In October 2017, we set the nylon mesh on the forest floor near each sampling tree to gather the fresh falling *L. formosana* litter for three weeks.

The collected litter was dried at 65 °C until it reached a stable weight. Subsequently, 20 g of *L. formosana* leaves or 7 g of *L. formosana* twigs was placed into each nylon mesh bag (25 × 25 cm, with 2 mm mesh size). In November 2017, six bags of leaves and six bags of twigs were buried beneath the humus layer around each sample tree after removing the existing litter near these trees. This setup resulted in a total of 216 litter bags, with 108 bags placed in the pure forest and 108 bags in the mixed forest.

After one, two, and three years of litter decomposition, two bags of leaves and two bags of twigs were taken to the laboratory in ice boxes. The litters from one leaf bag and one twig bag were dried at 65 °C until the weight stayed stable to determine the decomposition rate, which is the ratio of weight loss in the current year to the original weight. The first-year decomposition rate was calculated by

$$R=\frac{W0-W1}{W0},$$

while the second-year decomposition rate was calculated by

$$R=\frac{W1-W2}{W0},$$

and the third-year decomposition rate was calculated by

$$R=\;\frac{W2-W3}{W0}$$

The R stands for decomposition rate, while W1, W2, and W3 represent the litter constant weight after the drying process for the first, second, and third year, respectively. W0 is the original weight, 20 g for the leaves and 7 g for the twigs. The litter from the other two mesh bags was stored at +4 °C and −20 °C for enzyme activity measurements and DNA extraction, respectively.

### 2.2. DNA Extraction, Amplification of ITS2 Region, and Sequencing

Plant genomic DNA kits (Tiangen Biotech Company, Beijing, China) were used to extract the genomic DNA from each litter sample according to the manufacturer’s protocol [45]. Then the extracted DNA was tested on a NanoDrop ND-1000 spectrophotometer (Thermo Fisher Scientific, Waltham, MA, USA) for DNA quality and concentration. The fungal internal transcribed spacer 2 region (ITS2) was amplified by polymerase chain reaction (PCR) using the forward primer ITS1F (5′-CTTGGTCATTTAGAGGAAGTAA-3′) and the reverse primer ITS2 (5′-GCTGCGTTCTTCATCGATGC-3′) [46]. The triplicate 50 μL reaction mixture utilized for the PCR was provided by TransGen Biotech Company, Beijing, China, and comprised 25 μL of 2× Premix Taq, 1 μL of forward primer at a concentration of 10 μM, 1 μL of reverse primer at an equivalent concentration, 10 ng of template DNA, and an appropriate volume of nuclease-free water to reach the final reaction volume. A PCR control with sterile water instead of DNA was used to check for contamination. The PCR program included the following: 3 min at 95 °C for denaturation followed by 36 cycles of 30 s at 95 °C, 30 s at 55 °C for annealing, 45 s at 72 °C for elongation, and a final extension at 72 °C for 10 min. The product was analyzed using 2% agarose gel electrophoresis, then purified and quantified with a spectrophotometer. The amplicons underwent sequencing on an Illumina MiSeq platform (PE = 300) at Majorbio Company (Shanghai, China). The resulting raw sequence data were deposited in the National Center for Biotechnology Information (https://www.ncbi.nlm.nih.gov/) Sequence Read Archive under the project accession number PRJNA1056890, accessed on 26 December 2023.

### 2.3. Enzyme Activity Measurements

The activities of eight enzymes involved in carbon degradation, namely, β-xylosidase (XYL), β-d-glucuronidase (GLR), β-cellobiosidase (CEL), β-glucosidase (GLS), and laccase, along with enzymes implicated in nitrogen degradation (N-acetyl-glucosamidase, NAG), organic phosphorus degradation (phosphatase, PHO), and organic sulfur hydrolysis (sulfatase, SUL) were systematically measured and quantified after being stored at +4 °C for seven days after sample collection. The enzyme activities were quantified fluorometrically using 4-methylumbelliferone-linked (4-MUB) substrates [47]. For each sample, 0.45 g of litter was placed into three filter tubes and centrifuged following incubation in 600 μL of culture solution. The reaction was initiated by adding 200 μL of suspension containing 50 μL of the substrate to each well. The fluid was transferred to 5 mL tubes and adjusted to a volume of 3.5–4 mL with culture buffers to create enzyme-active samples. A negative control group was prepared by heating 1 mL of the sample fluid. Following the addition of fluorogenic substrate working solutions, the plates were incubated in the dark at 22 °C with shaking at 160 RPM for 15 min (for GLS and NAG) or 30 min (for all other enzymes except laccase). To terminate the reactions, 10 µL of 1 M NaOH was added. Fluorescence was measured using a Thermo Scientific Multiskan SkyHigh with excitation and emission filters set to 365 nm and 450 nm, respectively [48]. Enzyme activity was calculated based on the amount of MUB released from the litter samples over a specified period, expressed as nmol·g^−1^·h^−1^. For laccase measurements, ABTS solution was added to the sample plates, which were then incubated in the dark at 22 °C with shaking at 160 RPM for 60 min. The emission wavelength for laccase was set to 420 nm.

### 2.4. Quantitative Microbial Element Cycling

Quantitative Microbial Element Cycling (QMEC) employs a high-throughput quantitative PCR-based chip to assess and quantify the genetic potential of microbiota in transforming essential nutrients, including carbon, nitrogen, phosphorus, and sulfur [49]. The chip comprises 72 primer pairs targeting 64 microbial functional genes involved in carbon, nitrogen, phosphorus, and sulfur cycling, as well as methane metabolism. Twig litter from the third year of decomposition was selected for analysis. QMEC was conducted using a WaferGen Smart Chip Real-Time PCR system at Majorbio Company (Shanghai, China). Each primer set was run in triplicate, with a non-template negative control included in each run. The qPCR process included the first denaturation step at 95 °C for 5 min, followed by 40 cycles of denaturation at 95 °C for 30 s, annealing at 58 °C for 30 s, and extension at 72 °C for 30 s. Data quality control was performed using the Ct values for each gene, as analyzed with Canoco software 5 [49].

### 2.5. Sequence Data Processing and Statistical Analysis

Sequence processing was conducted on Mothur (v. 1.39.5), referring to the operating procedures described previously [50]. Initially, paired-end sequences were merged into contigs, followed by error correction using the chimera and PCR.seq commands. The corrected sequences were then assigned to operational taxonomic units (OTUs) with a 97% similarity threshold, employing the neighbor-joining algorithm [51]. Representative sequences from each OTU were classified using the UNITE database version 8.0, applying a bootstrap cutoff value of 80 [52]. Non-fungal sequences were eliminated using the ‘remove.lineage’ command. The sequence information after sequence denoising and quality filtering is shown in the supplementary table (Table S1). For data normalization, the smallest sample sizes with a sequence number were randomly subsampled for calculating the diversity indices and further analysis of fungal community structure. The OTUs were subsequently classified into ecological guilds: Saprotroph, Symbiotroph, and Pathotroph, based on FUNGuild, a functional annotation tool which uses a curated database to assign fungal taxa to guilds [53].

Across different decomposition years, one-way analysis of variance was utilized to assess significant differences in litter weight loss rates, enzyme activities, fungal community diversity, and fungal functional groups within the same litter type in SPSS v26.0 (Chicago, IL, USA). To compare these variables between different forests, the Kruskal–Wallis H test was utilized in SPSS v26.0. Spearman correlation analysis was utilized to test the correlation between diversity indices and enzyme activities in SPSS v26.0. Correlation heatmaps between genus abundance and enzyme activities and between genus abundance and gene abundance were generated on the Hiplot website (https://hiplot.com.cn, accessed on 24 September 2024). Graphpad Prism was utilized to visualize the results and to calculate R-square values and F-statistics within the framework of linear regression analysis. Principal coordinate analysis (PCoA) and PERMANOVA were employed to detect the significant differences in fungal community structure and fungal functional structure over three years, as well as the fungal functional gene community structure within twig litter from the third year, using Primer 7 software [54].

## 3. Results

### 3.1. Decomposition Rate and Enzyme Activities during Litter Decomposition

**Figure 1:**
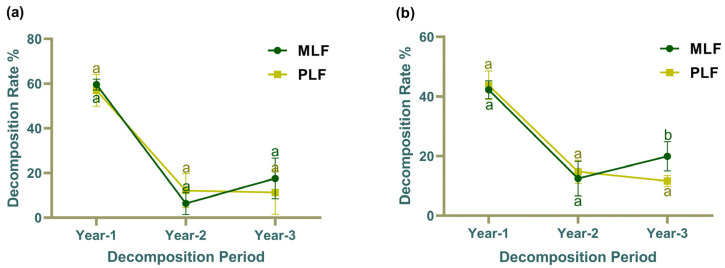
The litter decomposition rate of leaf (**a**) and twig (**b**) in mixed *Liquidambar formosana* forest (MLF) and pure *L. formosana* forest (PLF) over a 3-year period. Lowercase letters above the bars indicate statistically significant differences (*p* < 0.05) between the forests.

The decomposition rate of leaf litter did not exhibit significant differences between pure and mixed forest stands over the three-year period (Figure 1a). However, in the third year, the decomposition rate in twig litter was significantly higher in the mixed forest compared to the pure forest (*p* < 0.05) (Figure 1b). Both leaf and twig litters showed a similar trend over the 3-year period, with the highest decomposition rate in the first year (*p* < 0.05) and the lowest in the second year. Moreover, the leaf litter was decomposed faster than the twig litter in the first year (*p* < 0.05) (Table S2), but no difference in decomposition rates was observed between leaf and twig litters in the second and third years.

**Figure 2:**
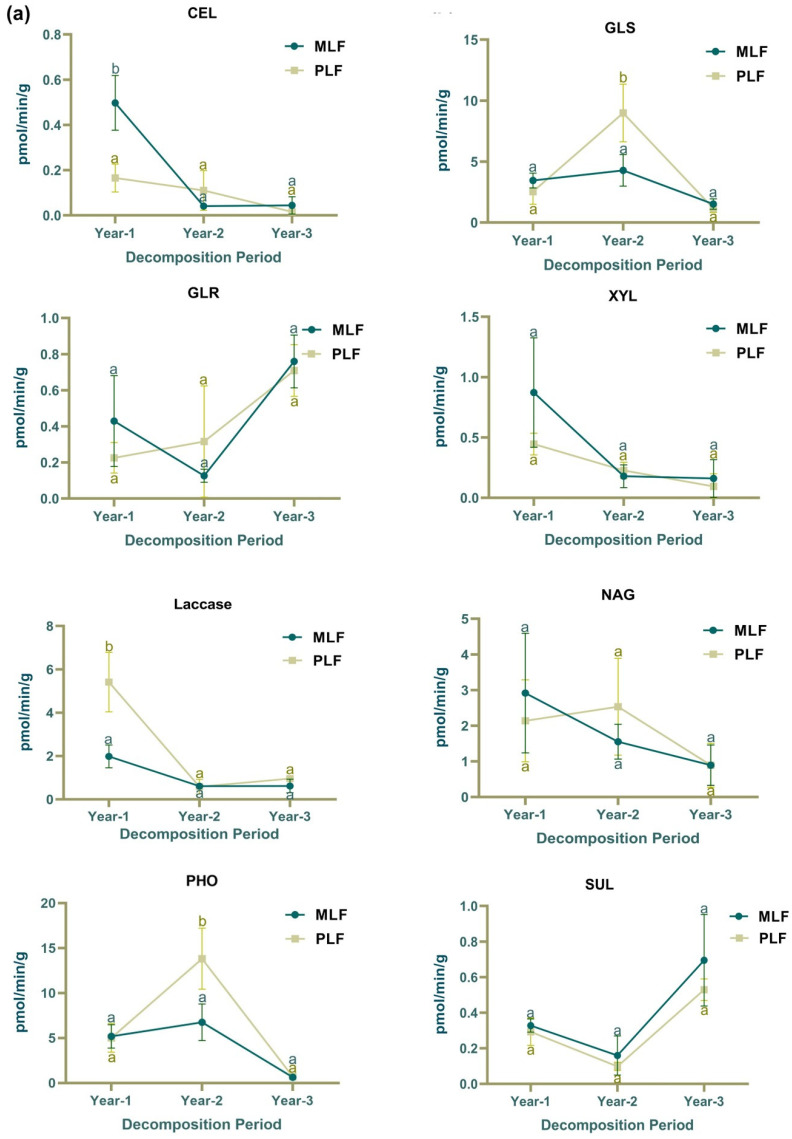

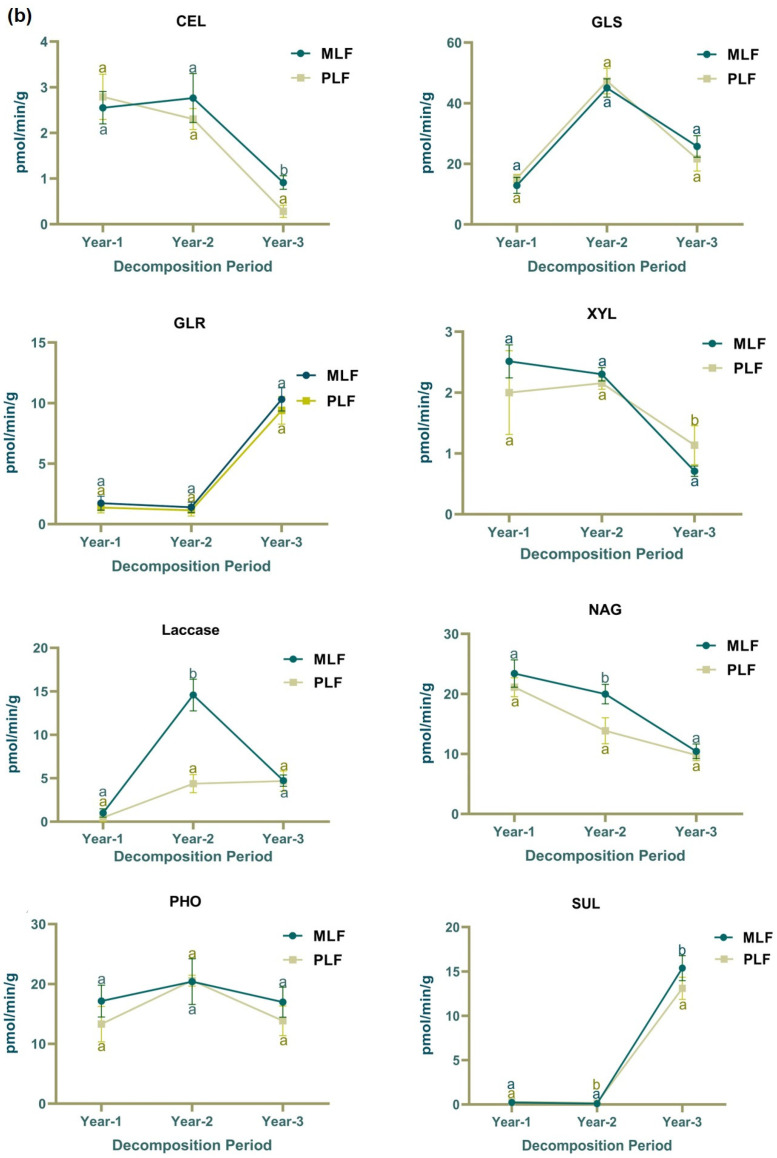
The activities of extracellular enzymes associated with carbon degradation, including β-cellobiosidase (CEL), β-glucosidase (GLS), β-D-glucuronidase (GLR), β-xylosidase (XYL), and laccase; nitrogen cycling (chitin decomposition N-acetyl-glucosamidase (NAG)); organic phosphorus decomposition (PHO); and sulfur hydrolysis (SUL) in leaf litter (**a**) and twig litter (**b**), respectively, in mixed *Liquidambar formosana* forest (MLF) and pure *L. formosana* forest (PLF) during the 3-year decomposition period. Lowercase letters above the bars indicate statistically significant differences (*p* < 0.05) between the forests.

In the first year of leaf decomposition, the activity of β-cellobiosidase (CEL) was significantly higher in the mixed forest (MLF) compared to the pure forest (PLF), whereas laccase activity was significantly higher in the pure forest (*p* < 0.05) (Figure 2a). During the second year, glucosidase (GLS) and phosphatase (PHO) activity in leaf litter were higher in the pure forest than in the mixed forest (*p* < 0.05) (Figure 2a). In the second year of twig decomposition, laccase and N-acetylglucosaminidase (NAG) activities were higher in the mixed forest, while sulfase (SUL) activity was higher in the pure forest (*p* < 0.05) (Figure 2b). In the third year of twig decomposition, CEL and SUL activity were higher in the mixed forest, while xylanase (XYL) activity was higher in the pure forest (*p* < 0.05) (Figure 2b).

In the mixed forest, the activities of CEL, XYL, and NAG in leaf litter decreased over the decomposition period (Table S3). In leaf litter, GLS and PHO activities increased in the second year and decreased in the third year, while SUL activity demonstrated the opposite pattern (*p* < 0.05) (Figure 2a). In twig litter, the activities of CEL, XYL, and NAG in the mixed forest also showed decreasing trends over time (Table S3), as did CEL and NAG activity in the pure forest (Table S3). GLS, laccase, and PHO activities increased in the second-year and decreased in the third-year twig decomposition (*p* < 0.05) (Figure 2b).

In the first-year decomposition, laccase activity was higher in leaf litter compared to twig litter, while all other enzymes except for SUL showed higher activities in twig litter (*p* < 0.05). In the second and third years, all tested enzymes showed higher activities in twig litter compared to leaf litter (*p* < 0.05)

The decomposition rate of leaf litter was positively correlated with the activities of CEL, XYL, and laccase (*p* < 0.01) (Table S4), while the decomposition rate of twig litter was positively correlated with CEL and NAG activities (*p* < 0.05) but negatively correlated with GLS and laccase activities (*p* < 0.01) (Table S5).

### 3.2. Fungal Community α-Diversity during Litter Decomposition

**Figure 3:**
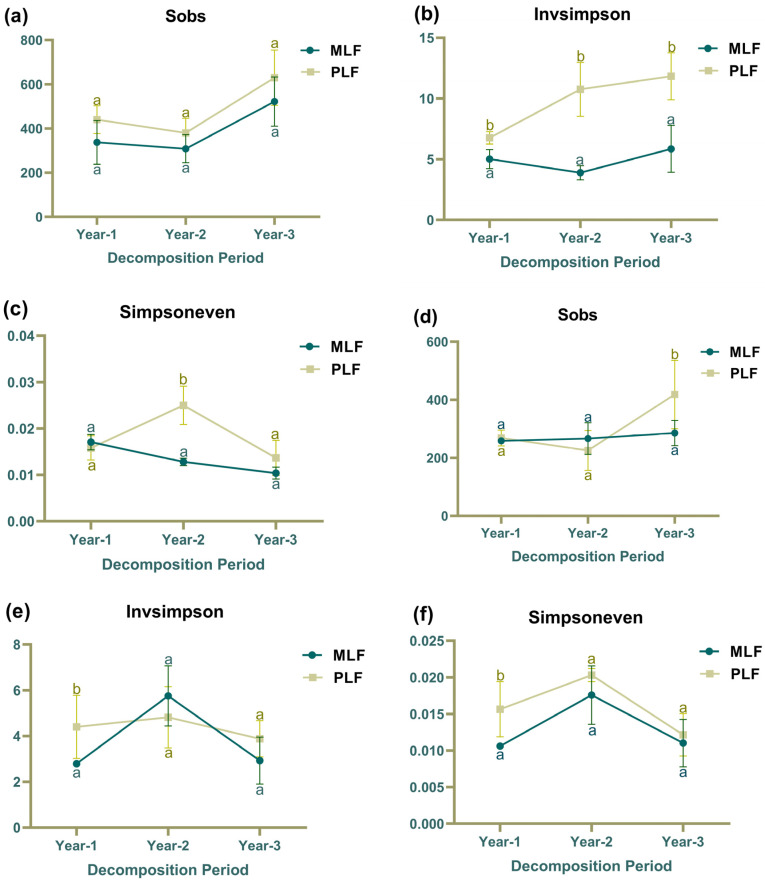
The fungal community richness (Sobs), fungal community diversity (Invsimpson), and fungal community evenness (Simpsoneven) within leaf litter (**a**–**c**) and twig litter (**d**–**f**) in mixed *Liquidambar formosana* forest (MLF) and pure *L. formosana* forest (PLF). Lowercase letters above the bars indicate statistically significant differences (*p* < 0.05) between the forests.

Fungal species richness (Sobs) within leaf litter did not differ between the pure and mixed forests over the three-year decomposition period (Figure 3a). However, the diversity (Inver Simpson index) was significantly higher in the pure forest compared to the mixed forest over the three years, with higher evenness (Simpsoneven) observed in the second year (*p* < 0.05) (Figure 3b,c). Within twig litter, the species richness was higher in the pure forest than in the mixed forest in the third-year decomposition (Figure 3d), and in the first-year decomposition, the diversity and evenness were higher in the pure forest as well (*p* < 0.05) (Figure 3e,f).

In the pure forest, fungal community diversity within leaf litter increased over the decomposition period (R^2^ = 0.63, F = 12.24) (Figure 3b). Fungal community evenness, however, exhibited decreasing trend in mixed forest (R^2^ = 0.8499, F = 39.62) (Figure 3c). Within twig litter, fungal community diversity and evenness in both forests experienced an increase followed by a decrease, while fungal community richness increased in the third year in the pure forest (*p* < 0.05) (Figure 3d,f).

In addition, compared to twig litter, leaf litter exhibited higher fungal community diversity in the first year of decomposition in both forests. However, in the third year, leaf litter harbored higher fungal species richness in the mixed forest (*p* < 0.05).

Fungal community richness demonstrated a positive correlation with GLR and SUL activities in both leaf and twig litters (Tables S4 and S5). During leaf decomposition, fungal community evenness showed a positive correlation with GLS, NAG, and PHO activities but a negative correlation with SUL activity (Table S2). Within twig litter, positive and negative correlations were observed between fungal community evenness with GLS and GLR activities, respectively (Table S5).

### 3.3. Fungal Composition at Taxonomic Level during Litter Decomposition

**Figure 4:**
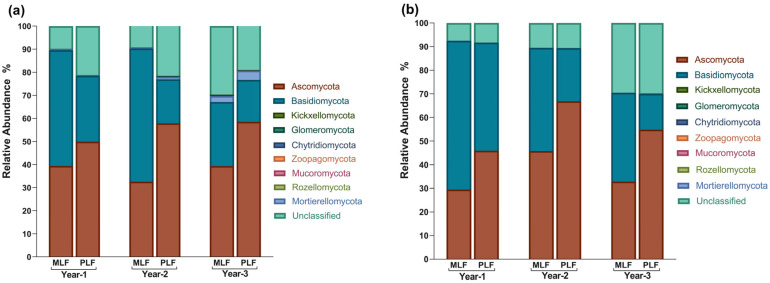

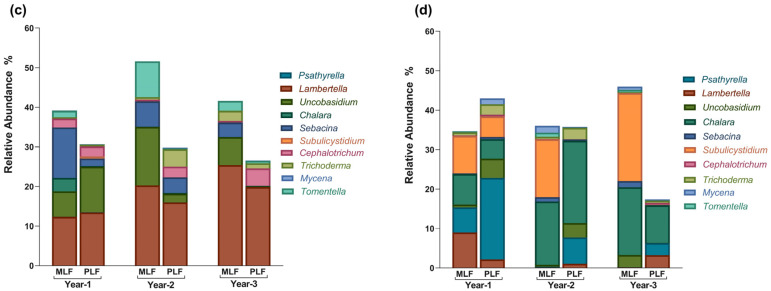
The relative abundance of fungal phyla and the top 10 genera in leaf litter (**a**,**c**) and twig litter (**b**,**d**) during decomposition in the mixed *L. formosana* forest (MLF) and pure *L. formosana* forest (PLF).

Ascomycota (48.28%) and Basidiomycota (38.94%) were the two most abundant phyla out of the nine detected phyla across the three-year decomposition, followed by Mortierellomycota (0.83%), Kickxellomycota (0.08%), and Glomeromycota (0.06%). Within leaf litter, the abundance of Ascomycota was higher in the pure forest compared to the mixed forest in the second and third years of decomposition (*p* < 0.05), while Basidiomycota had a higher abundance in the mixed forest during the first and second years (*p* < 0.05) (Figure 4a).

At the genus level, 359 genera were classified based on the identified OTU over three years. *Lambertella* and *Chalara* were the most abundant genera in leaf and twig litters, respectively. During leaf litter decomposition, *Chalara* and *Tomentella* were more abundant in the mixed forest compared to the pure forest in the first year and the first two years, respectively (*p* < 0.05) (Figure 4c). *Tomentella* and *Sebacina* were found to be richer in the second year and third year of twig decomposition in the mixed forest than in the pure forest (*p* < 0.05) (Figure 4c). Regarding leaf decomposition, the relative abundance of *Chalara* and *Uncobasidium* dropped in the second year (*p* < 0.05). *Lambertella*, *Sebacina*, *Tomentella*, *Uncobasidium*, and *Cephalotrichum* were richer in abundance in leaf litter, while *Chalara*, *Subulicystidium*, and *Psathyrella* held higher abundance in twig litter (*p* < 0.05).

**Figure 5:**
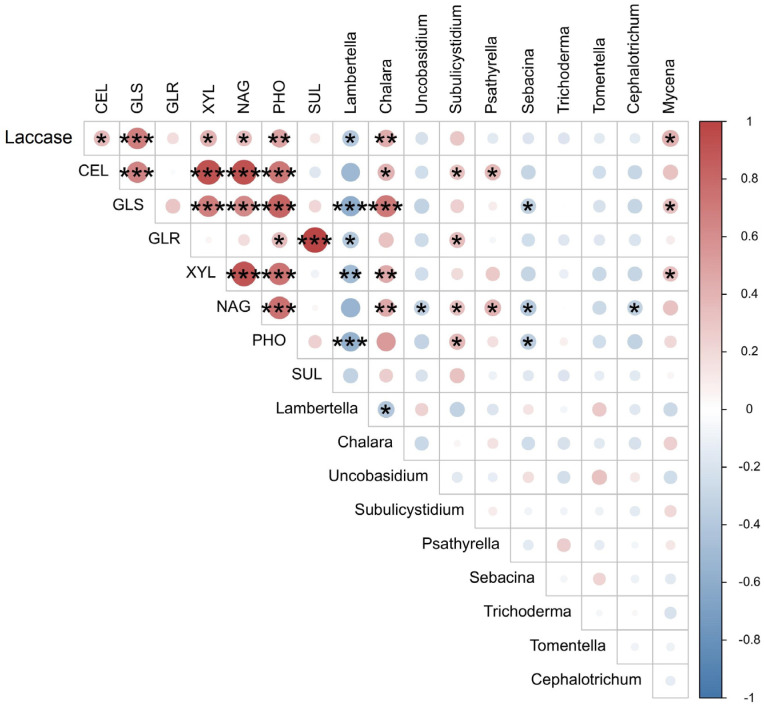
The correlation heatmap of the relative abundance of top ten genera and enzyme activities. A blue circle represents a negative correlation, and a red circle represents a positive correlation, with the color shade expressing the correlation value (from −1 to 1). A single asterisk (*) represents a *p*-value < 0.05, two asterisks (**) represent a *p*-value < 0.01, and three asterisks (***) represent a *p*-value < 0.001.

*Chalara* showed positive correlations with the activities of laccase, CEL, GLS, XYL, and NAG (*p* < 0.05) (Figure 5). *Subulicystidium* correlated positively with CEL, GLR, NAG, and PHO, whereas *Psathyrella* correlated positively with CEL and NAG. *Mycena* was found to build positive correlations with laccase, GLS, and XYL (*p* < 0.05) (Figure 5). The relative abundance of *Lambertella* was negatively correlated with the activities of laccase, GLS, GLR, XYL, and PHO (*p* < 0.05) (Figure 5). *Uncobasidium* and *Cephalotrichum* were negatively correlated with NAG, as was *Sebacina* with GLS, NAG, and PHO (*p* < 0.05) (Figure 5).

**Figure 6:**
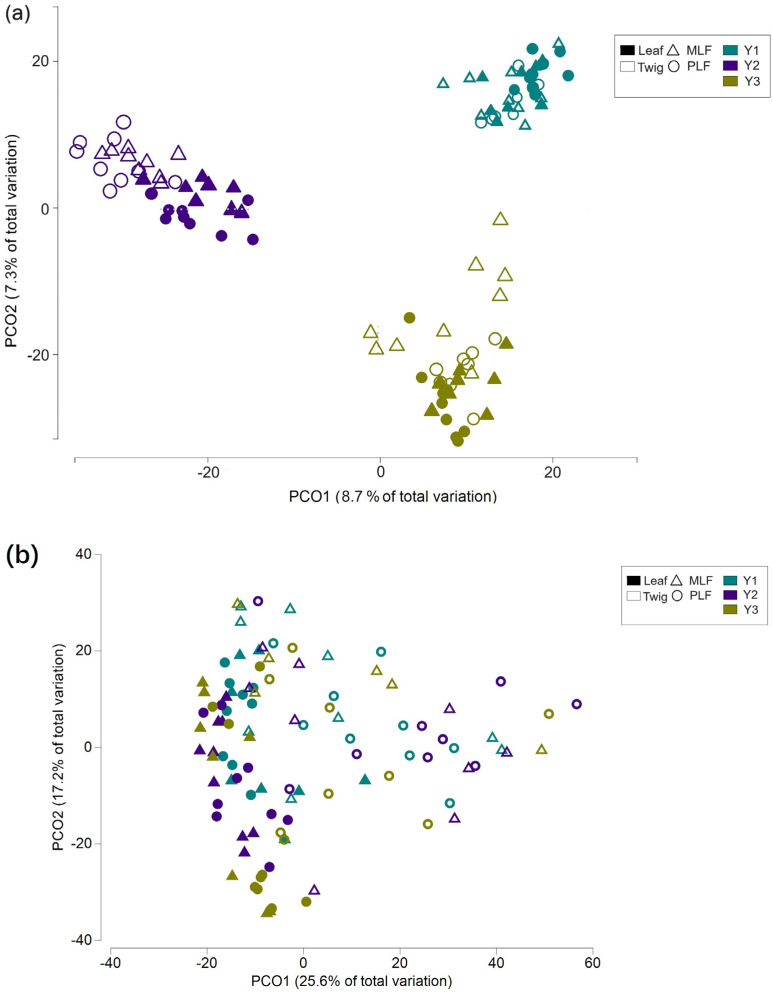

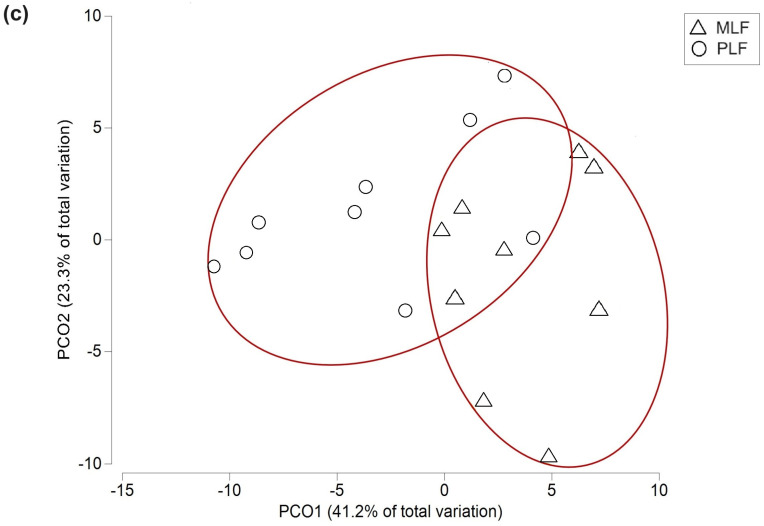
Principal coordinate analysis (PCoA) representing the fungal community structure (**a**), the fungal functional structure (**b**) within leaf and twig litters during the 3-year decomposition, and fungal genes within twig litter in the third decomposition year (**c**) in the mixed *Liquidambar formosana* forest (MLF) and the pure *L. formosana* forest (PLF). The red circles represent the separation of fungal genes between forest types.

The principal coordinate analysis (PCoA) constructed based on fungal OTU abundance indicated that the fungal community structures during the decomposition differed between the two forests within the same litter and between the two litters within the same forest (Figure 6a). The separation of fungal community structures was observed between different decomposition years (Figure 6a). These differences among community structures were subsequently confirmed by PERMANOVA results (*p* < 0.05 for each pair) (Table S6).

### 3.4. Fungal Functional Structure during Litter Decomposition

**Figure 7:**
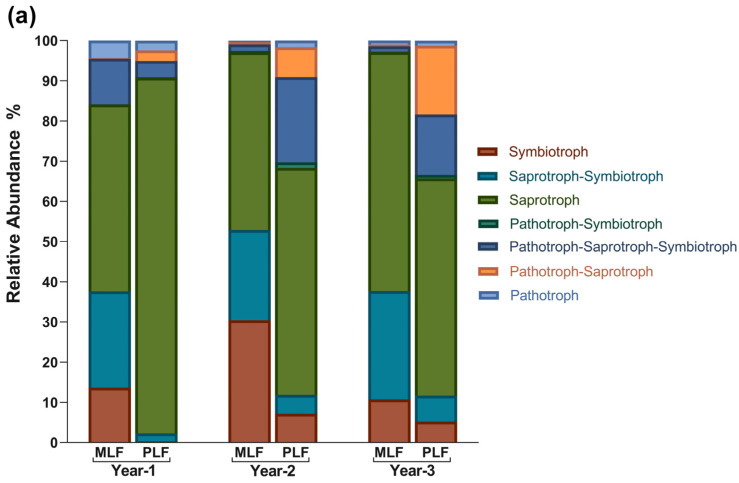

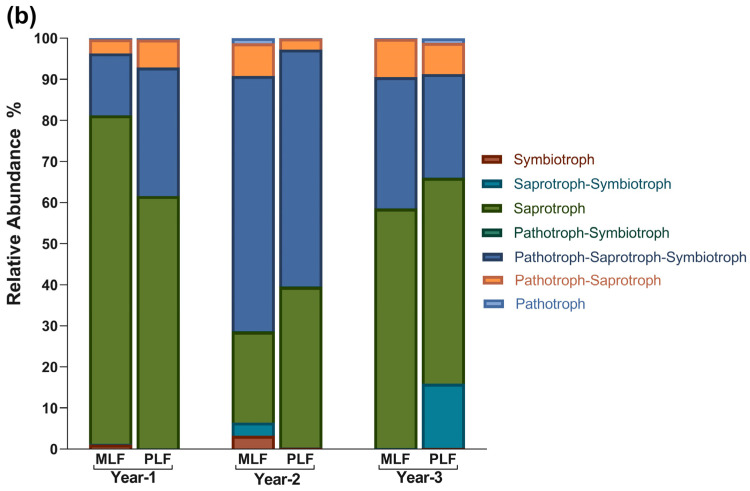
The relative abundance of fungal trophic modes based on FUNGuild analysis during the leaf litter decomposition (**a**) and twig litter decomposition (**b**).

In the FUNGuild analysis, 3983 OTUs (39.47%) were classified into seven trophic modes. Saprotroph was the most abundant group, covering 55.00% of the assigned OTUs, followed by Pathotroph–Saprotroph–Symbiotroph (23.15%), Saprotroph–Symbiotroph (8.82%), Symbiotroph (6.05%), Pathotroph–Saprotroph (5.51%), Pathotroph (1.20%), and Pathotroph–Symbiotroph (0.26%) (Figure 7).

Within leaf litter, Saprotroph–Symbiotroph and Symbiotroph had a higher abundance in the mixed forest during the decomposition period, while the pure forest had a higher abundance of Saprotroph, Pathotroph–Symbiotroph, and Pathotroph–Saprotroph (*p* < 0.05) (Figure S1a). Within twig litter, the abundance of Saprotroph in the mixed forest was higher in the first year but lower in the second year compared to the pure forest. Additionally, Symbiotroph had higher abundance in the mixed forest (*p* < 0.05) (Figure S1b).

Within leaf litter, the relative abundance of Symbiotroph, Pathotroph–Symbiotroph, and Pathotroph–Saprotroph increased significantly in the second year, while Saprotroph and Pathotroph decreased simultaneously (*p* < 0.05) (Figure S1a). In the third year of leaf decomposition, the relative abundance of Pathotroph–Symbiotroph and Symbiotroph dropped, while Saprotroph increased (*p* < 0.05) (Figure S1a). During twig decomposition, Symbiotroph, Pathotroph–Symbiotroph, and Pathotroph–Symbiotroph–Saprotroph all experienced an increase followed by decrease, peaking in the second year (*p* < 0.05) (Figure S1b). The relative abundance of Saprotroph, however, decreased and reached the lowest value in the second year of twig decomposition in both forests (*p* < 0.05) (Figure S1b).

In both forests, Symbiotroph, Saprotroph–Symbiotroph, and Pathotroph–Symbiotroph were more abundant within leaf litter during decomposition compared to twig litter, while Pathotroph–Symbiotroph–Saprotroph exhibited higher abundance within twig litter (*p* < 0.05).

PCoA based on the functional trophic modes showed distinct fungal trophic structures between the mixed and pure forests in each decomposition period, between the leaf and twig litters in each forest, and between the first-year decomposition and the subsequent period (Figure 6b). The differences in fungal trophic structures were later proved by PERMANOVA (*p* < 0.05 between all pairs) (Table S7).

### 3.5. Fungal Function Gene Structure in Twig Litter in the Third-Year Decomposition

**Figure 8:**
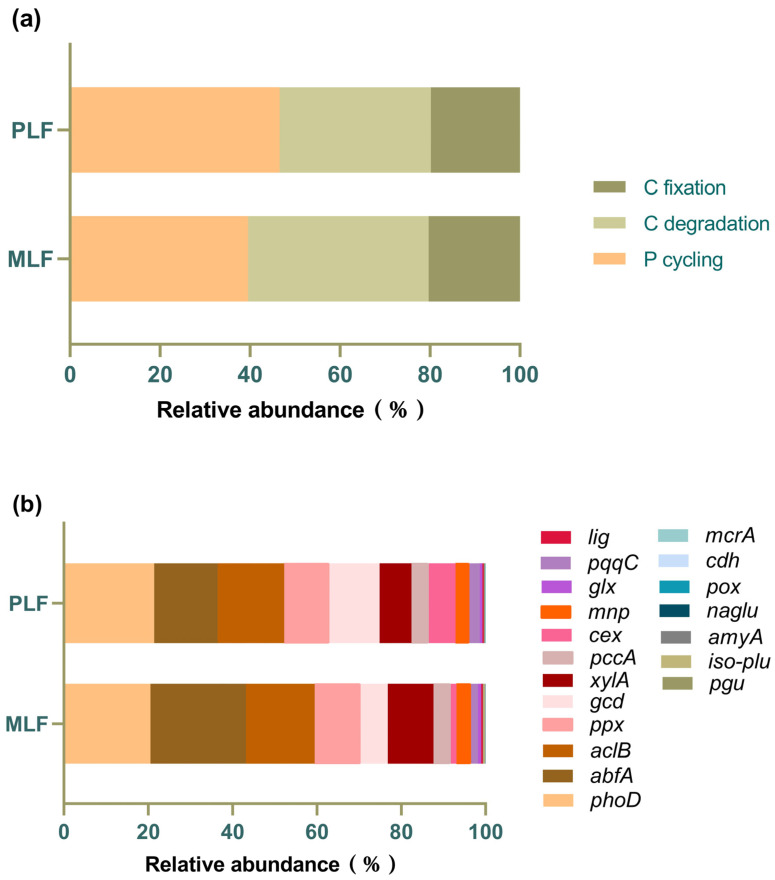
The relative abundance of fungal functional genes associated with C and P cycles (**a**) and individual fungal functional genes (**b**) in twig litter in the third-year decomposition in the mixed *Liquidambar formosana* forest (MLF) and the pure *L. formosana* forest (PLF).

A total of 19 functional genes associated with carbon degradation, carbon fixation, and phosphorus cycling were found in the twig litter. In both forests, genes participating in P cycling (43.22%) were the most abundant, followed by those involved in C degradation (36.76%) and C fixation (20.02%) (Figure 8a). Among individual genes, the top three were *phoD* (20.99%), *abfA* (18.72%), and *aclB* (16.01%), involved in carbon degradation, carbon fixation, and phosphorus cycling, respectively (Figure 8b).

The relative abundance of the gene *cex*, involved in carbon degradation, and the gene *gcd*, involved in phosphorus cycling, were both higher in the pure forest than those in the mixed forest (*p* < 0.05).

PCoA showed differences in the fungal functional gene structure in the twig litter between the mixed and pure forests (Figure 6c), which was further proved by PERMANOVA analysis (*p* < 0.001) (Table S8).

**Figure 9:**
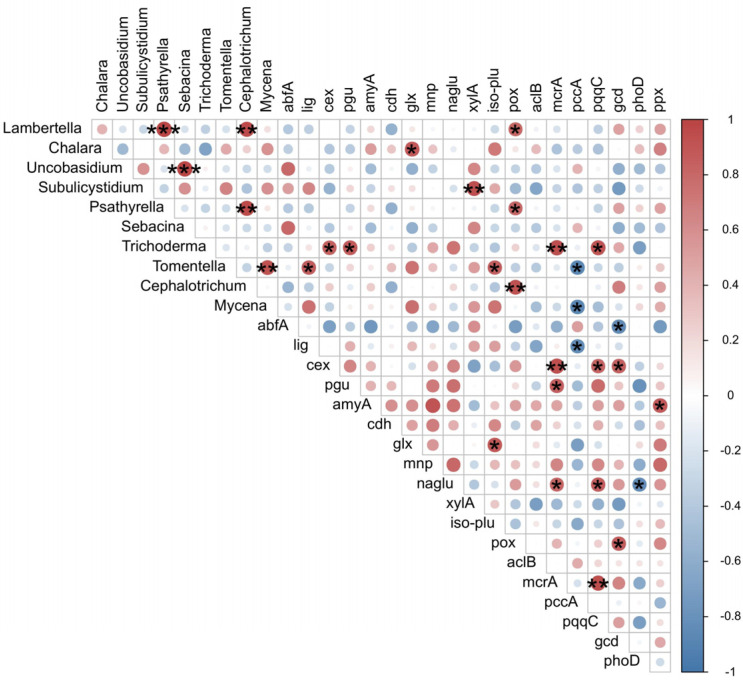
The correlation heatmap of the relative abundance of top ten genera and the relative abundance of genes. A blue circle represents a negative correlation, and a red circle represents a positive correlation, with the color shade expressing the correlation value (from −1 to 1). A single asterisk (*) represents a *p*-value < 0.05, two asterisks (**) represent a *p*-value < 0.01, and three asterisks (***) represent a *p*-value < 0.001.

*Trichoderma* was found to be correlated positively with *cex*, *pgu*, *mcrA*, and *pqqC*, whereas *Tomentella* was found to be correlated positively with *lig* and *iso-plu* (*p* < 0.05) (Figure 9). *Lambertella*, *Chalara*, and *Subulicystidium* were correlated positively with *pox*, *glx*, and *xylA*, respectively (*p* < 0.05) (Figure 9). The gene *pox* was positively correlated with both *Psathyrella* and *Cephalotrichum* (*p* < 0.05) (Figure 9). One negative correlation was found between *Tomentella* and *pccA* (*p* < 0.05) (Figure 9).

## 4. Discussion

The mixed forest exhibited a higher twig litter weight loss rate compared to the pure forest. This difference can be attributed to variations in the decomposer communities present in the two forests, as local decomposer communities significantly influence litter degradation [55]. Tree species impact litter decomposition not only through litter qualities but also by the specific conditions and decomposer communities in their forest floor [56]. Dominant plant species significantly affect both abiotic and biotic soil properties [57,58], which subsequently shape the litter decomposition by altering the decomposer assembly [59]. The litter decomposition rate decreased significantly from the first to the second year, reflecting the common temporal dynamic that the decomposition rate declines over time and stabilizes around a critical value [60]. Initially, microorganisms decompose small, readily available substances, while more complex macromolecular substances persist until the presence of some specific microorganisms which are capable of degrading them at a later stage [61,62]. The higher decomposition rate of leaf litter compared to twig in the first year suggests a difference in the initial qualities of readily degradable substances between leaf and twig litters.

In both leaf litter and twig litter, the mixed forest exhibited elevated β-cellobiosidase and N-acetyl-glucosamidase activities, whereas the pure forest showed higher activities of phosphatase, β-glucosidase, and β-xylosidase. These differences suggest that forest type does influence enzyme activity. Forests with specific tree species compositions impact soil properties through leaf litter and root exudates deposition [63]. Consequently, variations in tree species attributes lead to distinct soil environments [64], significantly affecting microbial activities through altering soil physical and chemical properties [65,66]. Enzyme activities, including β-cellobiosidase, β-xylosidase, and N-acetyl-glucosamidase, showed decreasing trends throughout decomposition in both litter types, consistent with the overall decline in decomposition rate. The activities of laccase within twig litter peaked in the second year, aligning with the previous research indicating high ligninolytic enzyme activity after 24 months of decomposition [67]. Laccases target aromatic moieties in lignin, with a preference for phenolic structures [68]. The peak in laccase activities suggests that significant decomposition of twig lignin occurred during the second year, indicating lignin removal following the decomposition of other components [67,69]. Higher enzyme activities were observed within twig litter compared to leaf, likely due to the presence of more refractory substances such as polyphenols, nitrogen, and lignin in twigs [70]. These substances attract a fungal community with higher enzyme production abilities. Strong positive correlations were found between the leaf litter decomposition rate and the activities of β-xylosidase, β-cellobiosidase, and laccase. β-xylosidase and β-cellobiosidase are extracellular hydrolytic enzymes related to hemicellulose and cellulose decomposition, respectively [71,72], breaking down the long chains of xylans and cellulose [68]. Thus, our results suggest that the loss of cellulose, hemicellulose, and lignin constitutes a significant proportion of the leaf litter mass loss in this study.

All three fungal α-diversity indices were higher in the pure forest. This suggests that tree species identity significantly influences the diversity and composition of the microbial community in organic soil [73], with conifer litter providing a poor substrate for microbial growth [22]. Consequently, the pure broadleaf forest likely offers a more favorable decomposing environment, assembling a decomposer community with higher diversity. Although more diverse microbe communities are hypothesized to enhance decomposition due to their ability to target various substrates and potentially include strong decomposers [74,75], increased diversity can also lead to competition or inhibition among fungal species [76]. This could explain the inhibited twig decomposition rate in the pure forest, attributed to adverse interactions within a diverse fungal community. The observed increase in fungal community species richness in the final year aligns with the peak richness of active fungi in logs at an advanced stage of decay [30]. Fungal community composition tends to shift rapidly with changing litter quality, driven by factors such as nutrient availability, other nutritional requirements, and the competitive abilities of individual taxa [32]. Competition likely diminishes as decomposition reaches its final phase, when nutrients become less available, leading to higher fungal species richness in the third year. Fungal species richness was positively correlated with β-d-glucuronidase and sulfatase activities, indicating that fungi vary in their enzyme production capabilities [77]. Community with more species tend to harbor higher enzyme activities, as a diverse community is more likely to include fungi that produce a broader range of enzymes targeting different components.

Within leaf litter, the pure forest exhibited a higher abundance of Ascomycota, while the mixed forest had more abundant Basidiomycota. Basidiomycota can synthesize enzymes essential for decomposition of complex polymers [78] and cause the loss of lignin and carbohydrates in variable proportions [79], making Basidiomycetes well suited for the more complex litter input in the mixed forest. In contrast, Ascomycota selectively decompose cellulose over lignin [32]. As the main wood decay fungi [80], Basidiomycota decreased in abundance along with twig decomposition as the decomposition process slowed. *Lambertella*, the most abundant genus in leaf litter, is known as a plant parasitic fungus capable of secreting lignin-degrading enzymes, including Mn-peroxidase and laccase [81,82]. Species of the genus *Chalara* predominantly thrive as litter saprotrophs, with a notable prevalence on coniferous litter [83], explaining its higher abundance in the mixed forest. Furthermore, *Chalara* built positive correlations with most of the enzymes involved in C degradation and was also positively correlated with the gene *glx*, responsible for lignin degradation [84]. Another study reported that *Chalara longipes* possessed a high content of genes involved in cell wall polysaccharide decomposition [85]. It thus could be inferred that *Chalara* could act as the key decomposers by facilitating enzyme production from a gene coding level. *Tomentella*, an ectomycorrhizal genus, can secrete extracellular enzymes that facilitate the decomposition of proteins, polysaccharides, and organic phosphorus compounds through root tips [86,87]. Another ectomycorrhizal genus, *Sebacina*, was also more abundant in the mixed forest [88]. The higher abundance of *Tomentella* and *Sebacina* in the mixed forest may be attributed to their ectomycorrhizal characteristics, allowing them to form mutualistic symbioses with various tree species [89].

Symbiotrophic and ectomycorrhizal fungi showed higher abundance in the mixed forest, whereas saprotrophic did not show a clear preference for either forest type. In forest ecosystems, nutrient constraints in soil have driven tree species to engage in mutualistic relationships with mycorrhizal fungi, which play a significant role in plant symbiosis [90]. High concentrations of polyphenols in conifer trees [91] potentially enhance the diversity of symbiotrophic fungi [92], leading to their prevalence in mixed forests. Evolutionarily, symbiotrophic fungi, compared to saprotrophs, have shifted from producing degrading enzymes to fulfilling new symbiotic functions, though they still retain some saprotrophic abilities such as producing cellobiohydrolases, ligninolytic MnP-ESD peroxidases, proteases, and laccases [93,94,95,96]. Ectomycorrhiza build associations with trees as the primary symbiosis [97] and use mycorrhizal hyphae extending from tree roots to reach the upper soil and litter layer and obtain essential nutrients, including inorganic and organic nitrogen and phosphate compounds [98,99]. The increased activity of N-acetyl-glucosamidase, involved in the nitrogen cycle and chitin degradation, could partly be due to the richer ectomycorrhiza presence in the mixed forest.

Microorganisms enhance nutrient cycling within ecosystems through diverse functional gene expression pathways [100]. After three-year decomposition, the fungal community in the pure forest emphasized phosphorus turnover more than carbon cycling at the gene level. This is logical given phosphorus’s essential and limiting role in soil fertility [101]. In our study, the most abundant genes in the mixed forest and pure forest were *abfA* and *phod*, respectively. The gene *abfA* encodes α-L-arabinofuranosidase (α-L-AFase), which catabolizes arabinoside and utilizes carbohydrates [102,103], while *phod* encodes alkaline phosphatase, participating in the mineralization of organic phosphorus [49]. In addition, the pure forest held higher abundance of *cex* and *gcd* genes, which are involved in cellulose hydrolysis [104] and solubilizing inorganic phosphorus, coding for quinoprotein glucose dehydrogenase [105,106], respectively. The divergence in gene structure between the two forests highlights the distinctiveness of fungal functional community at a deeper level. The correlation analysis of genus abundance and gene abundance revealed that most of the correlations were positive, indicating the contribution of the main occupying fungal taxa in mediating the practical nutrient cycling. The genus *Trichoderma*, which was reported to be a main source of biological beneficial agents in agriculture [107,108], was found to be correlated positively with the most genes, including cellulose hydrolysis gene *cex*, pectin degradation gene *pgu*, methane production gene *mcrA*, and *pqqC* [109,110,111]. Another genus, *Tomentella*, was positively correlated with *lig* and *iso-plu*, which are a lignin degradation gene and a starch degradation gene, respectively [112,113].

Based on OTU abundance, predicted functional modes, and fungal genes, distinct fungal communities and functional structures were formed in different forest types, emphasizing the influence of dominant tree species on the litter decomposing microbial community. Previous studies have reported a close association between aboveground plants and belowground fungal communities [48,114]. As plant characteristics change with the dominant tree species at a site, soil microbial communities can be influenced by microclimate variations, litter production (both above- and belowground), herbivore interactions, root exudate production, and symbiotic associations such as mycorrhizal fungi [114]. In our study, the drying process removed previously colonized microbes, making the litter microbial community closely associated with the soil microbial community, as soil microbes serve as a primary source of litter-degrading microorganisms [115]. In the Abitibi region, diverse microbial communities, both functionally and genetically distinct, were also observed in forest stand floors dominated by different tree species [116]. Microbial communities related to litter decomposition are shaped by substrate characteristics, which determine the capacity of different taxa to obtain and utilize distinct structural substrates [14,31,61]. Therefore, the different decomposing conditions provided by various forest types shape the distinct microbial communities. The first-year decomposition exhibited a distinct functional community structure compared to the following two years, corresponding with the dynamic pattern of the decomposition rate. This demonstrates the interaction between fungal community structure and litter decomposition patterns. As expected, twig and leaf harbored separate fungal communities, reflecting the sensitivity of fungal communities to litter qualities [117].

## 5. Conclusions

The litter decomposition rate differed significantly in the third year of twig decomposition, which could be supported by the different higher enzyme activities in the mixed *L. formosana* forest (β-cellobiosidase and N-acetyl-glucosamidase) and the pure forest (phosphatase, β-xylosidase, and β-glucosidase). Fungal community species richness, diversity, and evenness were higher in the pure forest owing to its more favorable condition. The genus *Chalara* and *Trichoderma* were positively correlated with enzyme activities and gene abundance, suggesting their key roles in litter decomposition. Functionally, symbiotrophic fungi and ectomycorrhizal fungi were more abundant in the mixed forest, supporting the litter decomposition in a potential way. In the third year of twig decomposition, genes associated with phosphorus cycling were the most abundant. Different forest types harbored distinct fungal community structure at community, functional, and gene levels. These findings suggest that forest types strongly influence litter decomposition by recruiting different fungal communities. Additionally, decomposition time and litter quality affect the decomposition by shifting substrate characteristics.

## Data Availability

The data analyzed in this study are accessible upon request from the corresponding author. Additionally, the raw sequence data have been deposited in the NCBI Sequence Read Archive (SRA) and are available under the project accession number PRJNA1056890.

## Supplementary Materials

The following supporting information can be downloaded at: https://www.mdpi.com/article/10.3390/jof10100690/s1. Figure S1: The relative abundance of the main trophic modes of Symbiotroph, Saprotroph-Symbiotroph, Saportroph, Pathotroph-Symbiotroph, Pathotroph-Saprotroph-Symbiotroph, Pathotroph-Saprotroph, Pathotroph in leaf litter (a) and twig litter (b) in the mixed *Liquidambar formosana* forest (MLF), and the pure *L. formosana* forest (PLF). Lowercases above represent the significant difference (*p* < 0.05) between different forests; Table S1: Sequences information after denoising and quality filtering; Table S2: Independent-samples Kruskal-Wallis tests showing the difference of decomposition rate from different litter qualities (leaf and twig) in the same forest; Table S3: R square and F value showing the decreasing trend of the enzyme activities along the three-year decomposition; Table S4: Correlation analysis of decomposition rate and fungal diversity indices with enzyme activities within leaf litter; Table S5: Correlation analysis of fungal diversity indices with enzyme activities within twig litter; Table S6: PERMANOVA results showing the difference in fungal community structure; Table S7: PERMANOVA results showing the difference in fungal community functional structure; Table S8: PERMANOVA results showing the difference in fungal gene structure.
