# Supplementary material for: Dominant Tree Species and Litter Quality Govern Fungal Community Dynamics during Litter Decomposition
Source: J Fungi (Basel). 2024 Oct 3;10(10):690. doi: 10.3390/jof10100690 (PMC11508307; doi:10.3390/jof10100690)
Supplement: Supplementary file 1 [file jof-10-00690-s001.zip › jof-3222639-supplementary.pdf]

(a)

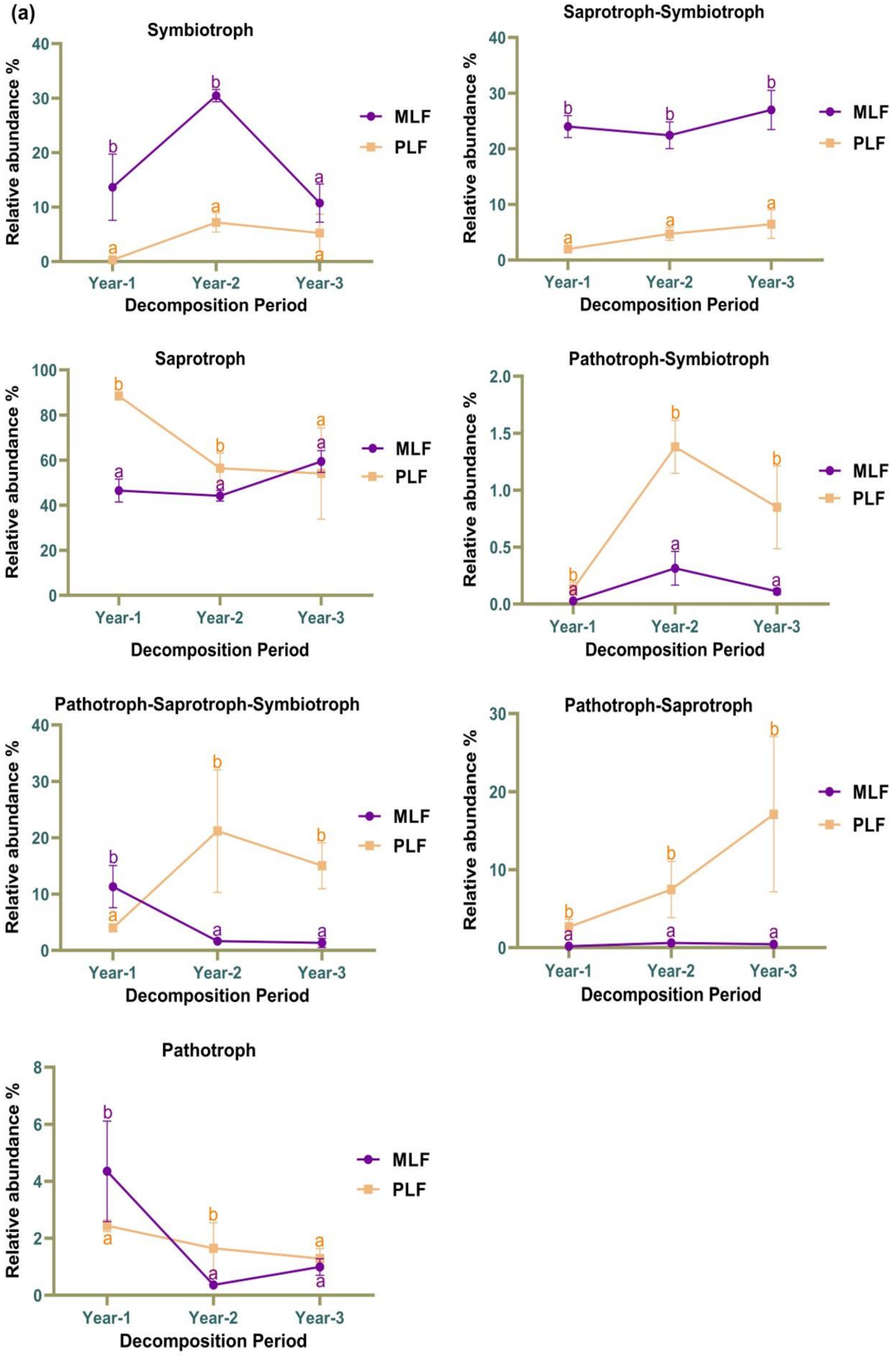
Symbiotroph
Saprotroph-Symbiotroph
Saprotroph
Pathotroph-Symbiotroph
Pathotroph-Saprotroph-Symbiotroph
Pathotroph-Saprotroph
Pathotroph
Relative abundance %
Decomposition Period
Year-1
Year-2
Year-3
MLF
PLF

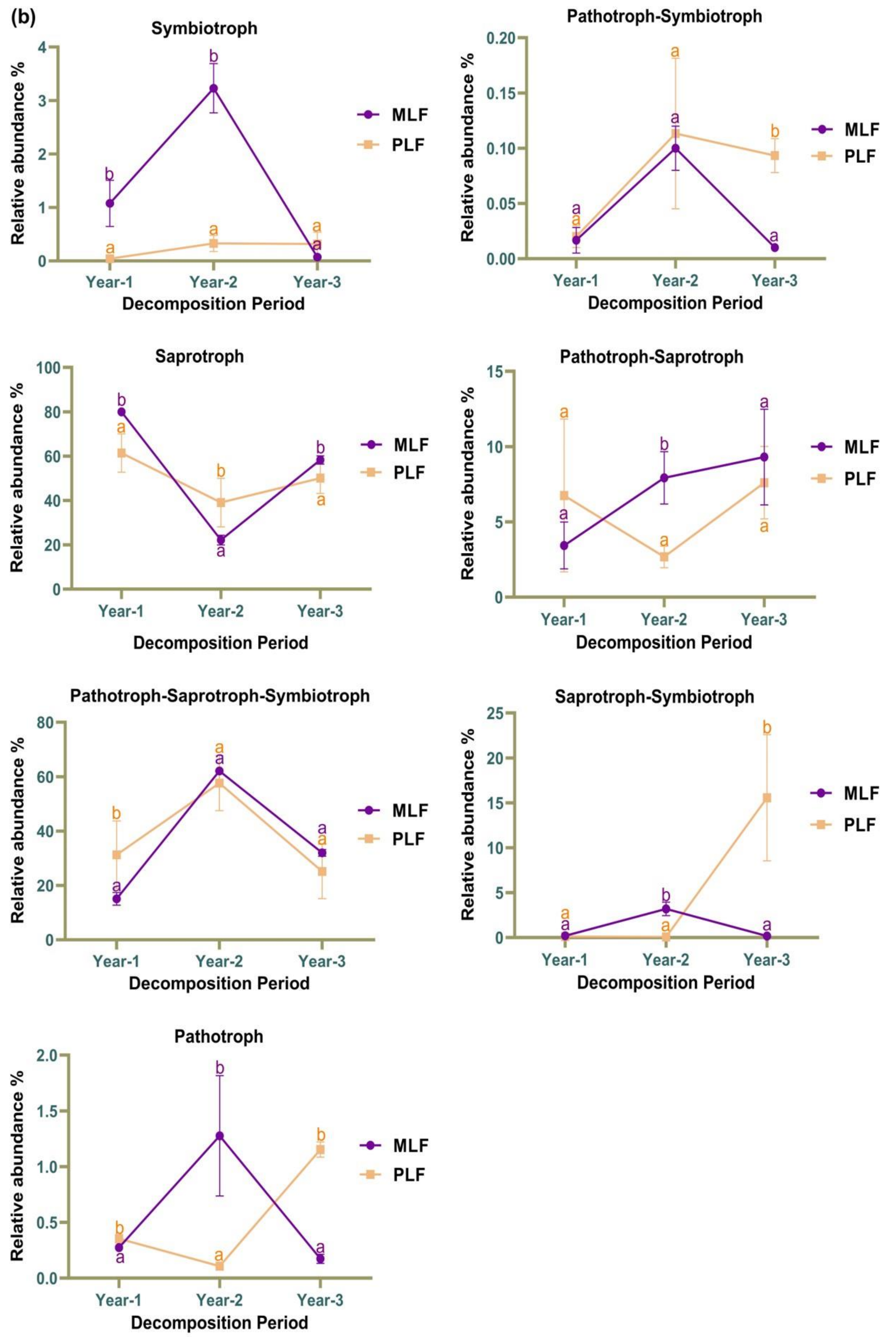

Fig. S1. The relative abundance of the main trophic modes of Symbiotroph, Saprotroph-Symbiotroph, Saportroph, Pathotroph-Symbiotroph, Pathotroph-Saprotroph-Symbiotroph, Pathotroph-Saprotroph, Pathotroph in leaf litter (a) and twig litter (b) in the mixed *Liquidambar formosana* forest (MLF), and the pure *L. formosana* forest (PLF). Lowercases above represent the significant difference ($P < 0.05$) between different forests.

Table S1. Sequences information after denoising and quality filtering.

Decomposition period	Number of sequences	Average number of sequences per sample (mean ± standard deviation)	Range of sequences	Range of Good's coverage
Year-1	3,583,631	99,545 ± 38,573	14,921~194,600	0.9832~0.9983
Year-2	3,519,175	97,755 ± 56,777,	13,839~359,224	0.9840~0.9970
Year-3	3,355,991	95,885 ± 35,595	31,764~200,269	0.9881~0.9983

Table S2. Independent-samples Kruskal-Wallis tests showing the difference of decomposition rate from different litter qualities (leaf and twig) in the same forest. MLF: the mixed *Liquidambar formosana* forest.

Decomposition period	Forest type	Sig.	Pattern
Year-1	MLF	**<0.05**	Leaf > Twig

Table S3. R square and F value showing the decreasing trend of the enzyme activities along the three-year decomposition.

Enzyme	Forest type	Litter quality	R square	F-value
CEL	MLF	Leaf	0.69	15.64
XYL	MLF	Leaf	0.52	7.54
NAG	MLF	Leaf	0.47	6.16
CEL	MLF	Twig	0.57	9.42
XYL	MLF	Twig	0.81	30.11
NAG	MLF	Twig	0.87	47.09
CEL	PLF	Twig	0.84	36.43
NAG	PLF	Twig	0.90	63.34

CEL: β-cellobiosidase, XYL: β-xylosidase, NAG: N-acetyl-glucosamidase. MLF: the mixed *Liquidambar formosana* forest, PLF: the pure *L. formosana* forest.

Table S4. Correlation analysis of decomposition rate and fungal diversity indices with enzyme activities within leaf litter.

		CEL	GLS	GLR	XYL	NAG	PHO	SUL	ABTS
Decomposition	Coefficient	0.729	-0.216	-0.154	0.738	0.386	-0.120	0.020	0.716
rate	*P*	**<0.01**	0.404	0.555	**<0.01**	0.126	0.645	0.939	**<0.01**
sobs	Coefficient	-0.381	-0.407	0.482	-0.396	-0.548	-0.53	0.672	-0.062
	P	0.119	0.094	**<0.05**	0.104	**<0.05**	**<0.05**	**<0.01**	0.807
invsimpson	Coefficient	-0.363	0.124	0.315	-0.376	-0.202	0.003	0.164	-0.183
	P	0.167	0.647	0.235	0.152	0.454	0.990	0.544	0.497
simpsoneven	Coefficient	0.250	0.817	-0.264	0.237	0.498	0.766	-0.538	0.075
	P	0.318	**<0.01**	0.290	0.343	**<0.05**	**<0.01**	**<0.05**	0.768

Sobs: fungal community species richness, invsimpson: fungal community diversity, simpsoneven: fungal community evenness. CEL: β-cellobiosidase, GLS: β-glucosidase, GLR: β-D-glucuronidase, XYL: β-xylosidase, ABTS: laccase, NAG: N-acetyl-glucosamidase, PHO: acid phosphatase and SUL: sulfatase.

Table S5. Correlation analysis of fungal diversity indices with enzyme activities within twig litter.

		CEL	GLS	GLR	XYL	NAG	PHO	SUL	ABTS
Decomposition	Coefficient	0.479	-0.712	-0.378	0.322	0.641	-0.261	-0.398	-0.665
rate	*P*	**<0.05**	**<0.01**	0.122	0.192	**<0.01**	0.296	0.102	**<0.01**
sobs	Coefficient	-0.587	-0.226	0.557	-0.377	-0.387	-0.383	0.523	0.009
	P	**<0.05**	0.368	**<0.05**	0.123	0.112	0.117	**<0.05**	0.973
invsimpson	Coefficient	0.220	0.480	-0.372	0.111	0.100	0.071	-0.343	0.397
	P	0.412	0.060	0.156	0.683	0.712	0.793	0.193	0.128
simpsoneven	Coefficient	0.325	0.673	-0.516	0.286	0.137	0.284	-0.464	0.281
	P	0.189	**<0.01**	**<0.05**	0.249	0.587	0.254	0.052	0.258

Sobs: fungal community species richness, invsimpson: fungal community diversity, simpsoneven: fungal community evenness. CEL: β-cellobiosidase, GLS: β-glucosidase, GLR: β-D-glucuronidase, XYL: β-xylosidase, ABTS: laccase, NAG: N-acetyl-glucosamidase, PHO: acid phosphatase and SUL: sulfatase.

Table S6. PERMANOVA results showing the difference in fungal community structure.

Pairs	*P*-value	*F*-value
MLF vs PLF	**<0.05**	2.53
Y1 vs Y2	**<0.05**	5.21
Y2 vs Y3	**<0.05**	4.97
Y1 vs Y3	**<0.05**	4.58
Leaf vs Twig	**<0.05**	5.61

MLF: the mixed *Liquidambar formosana* forest, PLF: the pure *L. formosana* forest.

Table S7. PERMANOVA results showing the difference in fungal community functional structure.

Pairs	*P*-value	*F*-value
MLF vs PLF	**<0.05**	1.86
Y1 vs Y2	**<0.05**	1.57
Y2 vs Y3	0.259	1.11
Y1 vs Y3	**<0.05**	1.76
Leaf vs Twig	**<0.05**	4.03

MLF: the mixed *Liquidambar formosana* forest, PLF: the pure *L. formosana* forest.

Table S8. PERMANOVA results showing the difference in fungal gene structure.

Pairs	*P*-value	*F*-value
MLF vs PLF	**<0.001**	2.27

MLF: the mixed *Liquidambar formosana* forest, PLF: the pure *L. formosana* forest.
